# Ambulatory surgical management of most displaced tibial tubercle fractures in children is safe and efficient

**DOI:** 10.1177/18632521231214317

**Published:** 2023-11-16

**Authors:** Brian Kendrick Zukotynski, Danielle Brown, Kellyn Hori, Mauricio Silva

**Affiliations:** 1 Department of Orthopaedic Surgery, University of California, Los Angeles, Santa Monica, CA, USA; 2 The Luskin Orthopaedic Institute for Children, Los Angeles, CA, USA

**Keywords:** Tibial tubercle fracture, outpatient surgery, fracture surgery

## Abstract

**Purpose:**

The purpose of this study is to compare the outcome of patients with displaced tibial tubercle fractures treated surgically who spent one or more nights in the hospital after surgery with that of patients treated in an ambulatory setting with no perioperative hospitalization. We hypothesized that tibial tubercle fractures have a low rate of complications and that most patients do well without an overnight hospital stay for observation.

**Methods:**

We retrospectively reviewed all pediatric tibial tubercle fractures treated operatively by a single surgeon over a 13.5-year period. Fractures treated in an inpatient setting, defined as at least one night of overnight hospitalization postoperatively, were compared with fractures treated in an ambulatory setting with no perioperative hospitalization.

**Results:**

Seventy-one fractures in 70 patients were analyzed. All fractures were treated with open reduction and internal fixation with unicortical screws. Thirty-five fractures (49.3%) were fixed in an ambulatory setting, while 36 (50.7%) were inpatient. There were no significant differences between inpatient demographics (age, gender, body mass index, fracture type). Average operative time was significantly longer in the inpatient group compared with the ambulatory group (97.8 min versus 58.8 min, p < 0.001). There was no significant difference in the incidence of complications between inpatient and ambulatory groups (25.0% versus 11.4%, p = 0.22). No cases of compartment syndrome were noted.

**Conclusion:**

Ambulatory surgical treatment of select tibial tubercle fractures with same-day discharge is safe and efficient. Not all patients with surgically treated tibial tubercle fractures need to stay overnight in the hospital.

## Introduction

Tibial tubercle fractures account for less than 3% of epiphyseal injuries and only 1% of all physeal injuries in children and adolescents despite the frequency of chronic injury in this anatomic region.^1–3^ These injuries typically occur in adolescent males approaching skeletal maturity when participating in athletic activities, particularly those that involve jumping.^2,4,5^ For these patients, powerful contraction during take-off or rapid passive flexion of the knee against a contracting quadriceps muscle during landing result in avulsion of the proximal tibial epiphysis from the tibial metaphysis. Management has long been based on radiographic classification and degree of displacement, with a general trend toward operative management of displaced fractures,^6–10^ though evidence-based recommendations surrounding perioperative management are limited secondary to the low incidence of these fractures.

For many straightforward pediatric surgeries, treatment in an ambulatory setting with no need for perioperative hospitalization provides a quick, safe, and cost-effective alternative to inpatient surgery.^11–14^ Most literature regarding pediatric tibial tubercle fractures, however, describes postoperative hospital admission for observation as the default course of treatment for routine fracture repair.^10,15^ While the complication rate associated with tibial tubercle fractures ranges from 10% to 28%,^2,10^ most of the described complications are chronic in nature, including hardware irritation and tenderness over the tibial tubercle, with the majority of patients having excellent functional and radiographic outcomes.^5,16^

Given the low rate of acute complications, it is possible that a select group of pediatric patients with displaced tibial tubercle fractures do not require routine hospitalization or overnight observation following surgical fracture repair. However, one barrier to treatment of tibial tubercle fractures in an ambulatory setting, with no need for perioperative hospitalization, is concern regarding the development of compartment syndrome.^17–19^ The tibial tubercle is closely associated with the fascia of the anterior compartment near a fan-shaped group of vessels originating from the anterior recurrent tibial artery,^20^ and these vessels are prone to bleeding into the anterior compartment when damaged.^21^ Elevated pressures in posterior and lateral compartments of the knee have also been described in association with tibial tubercle fractures.^22^ The reported incidence of suspected compartment syndrome is as high as 10%–20%,^8,23^ with many surgeons electing to perform prophylactic fasciotomy at any sign of a firm anterior compartment.^10,19^ However, actual confirmed instances of compartment syndrome are rarer and are usually associated with more extensive injuries.^24–27^ A recent systematic review of 336 tibial tubercle fractures found a 4% incidence of preoperative compartment syndrome and a 0% incidence of postoperative compartment syndrome.^2^ Given that the rate of associated compartment syndrome may be lower than what has been previously described, selective ambulatory management may be an option for tibial tubercle fractures.

The purpose of this study is to report the outcomes of a large, single-surgeon series of tibial tubercle fractures treated surgically at our institution and to compare the outcome of patients who spent one or more nights in the hospital after surgery with that of patients treated in an ambulatory setting with no perioperative hospitalization. We hypothesized that tibial tubercle fractures have a low rate of complications and that most patients do well without an overnight hospital stay for observation.

## Methods

**Figure 1.**
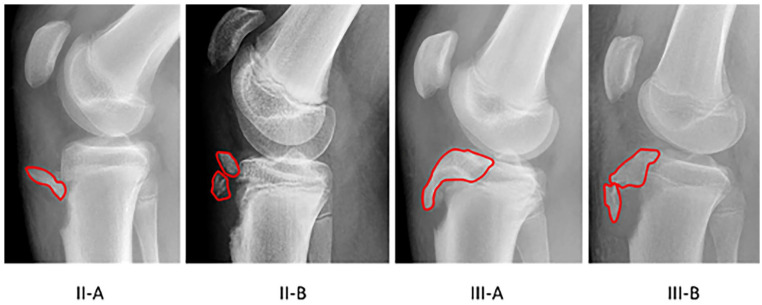
Representative preoperative lateral X-rays demonstrating the modified Ogden classification of tibial tubercle fractures. In a type I fracture (not depicted), the fracture line is through the secondary ossification center. In a type II fracture, the fracture occurs through the junction of the proximal tibia and tubercle ossification centers. Type II fractures can be either not comminuted (II-A) or comminuted (II-B). A type III fracture is a true Salter-Harris type III injury that is intra-articular. In subtype III-A, the tubercle and the anterior part of the proximal tibial epiphysis form a single unit. In subtype III-B, the fragment is comminuted, and the site of fragmentation is at the junction of the ossification centers of the proximal end of the tibia and the tubercle. A type IV fracture (not depicted) extends posteriorly through the entire proximal tibial physis, creating an avulsion of the entire epiphysis.^7,28^

Following approval from our Institutional Review Board, all tibial tubercle fractures treated surgically by the senior author between 1 December 2005 and 1 September 2019 were reviewed. Patients were excluded for incomplete clinical or radiographic records. All clinical and radiographic records were reviewed. Demographic data collected included patient age and gender, body mass index (BMI), and medical comorbidities. Injury-related variables examined included time to presentation and surgery, side of injury, injury mechanism and activity, and fracture classification and characteristics (degree of displacement, presence of intra-articular involvement, concomitant injuries including patellar sleeve avulsion and meniscal injury). The fracture was classified on preoperative plain lateral radiographs using the modified Ogden et al.^7,28^ classification (Figure 1). The amount of displacement was classified as minimal (<2 mm), moderate (2–20 mm), or severe (>20 mm). Comminution was defined intraoperatively as two or more fracture fragments.

Operative variables collected included surgical setting (patients who spent one or more nights in the hospital after surgery (inpatient or Group 1) versus patients treated with no perioperative hospitalization (outpatient or Group 2)), number of screws, screw configuration, whether or not fasciotomy was performed, and surgical time. Before December 2016, all fractures were managed in a general hospital. From 2005 through 2013, all fractures were managed in an inpatient fashion (n = 29). From 2014 through 2016, at the discretion of the treating surgeon based on injury severity and operating room availability, some fractures were managed in an inpatient fashion (n = 4) while others were outpatient (n = 16). After 2016, only three fractures were managed in a general hospital with an inpatient stay, while the majority of fractures were managed in a stand-alone orthopedic ambulatory surgery center, in an outpatient fashion (n = 19). Therefore, there were 36 (50.7%) fractures in Group 1 and 35 (49.3%) fractures in Group 2. Operative time was defined from incision until completion of skin closure and did not include anesthesia time. Postoperative data collected for our analysis included duration of hospitalization, follow-up duration, type and duration of immobilization, range of motion at final follow-up, and complications. Patients were classified as having stiffness if they had an extension lag ≥5° and/or flexion ≤110°.

The demographic variables, fracture characteristics, surgical time, and complications were compared between groups. For numerical variables, a nonparametric Mann–Whitney Wilcoxon test or one-way analysis of variance (ANOVA) was utilized. A Pearson correlation coefficient was used to assess for linear associations. A Fisher’s exact test was used for categorical variables. Significance was defined as p < 0.05. All statistical analysis was performed using GraphPad Prism Statistics/Data Analysis software (GraphPad Software Inc., La Jolla, CA).

## Results

From December 2005 to September 2019, 70 patients with 71 tibial tubercle fractures underwent surgical treatment by a single attending surgeon at our institution. Of the 70 patients, 46 (65.7%) sustained left-sided fractures, while 23 (32.9%) sustained right-sided fractures (p = 0.62; Table 1). One patient (1.4%) sustained bilateral fractures.

**Table 1.** Patient and fracture characteristics: inpatient versus outpatient surgery.

	Group 1 (n = 35 patients, 36 fractures)^a^	Group 2 (n = 35 patients, 35 fractures)	p-value
Age (years)^b^	14.6 (14.2–15.1)	14.6 (14.2–15.0)	0.95
Sex (M/F) (%)	94.4/5.6	91.4/8.6	0.67
BMI^b^	29.0 (26.7–31.4)^c^	26.9 (24.8–29.1)	0.22
Age-sex-matched BMI percentile^b^	92.3 (86.3–98.3)^c^	79.9 (68.9–90.9)	0.27
Side of injury (L/R) (%)	69.4/30.6	62.9/37.1	0.62
Injury classification (%)	–	–	0.08
Type I	8.3	5.7	–
Type II	25.0	51.4	–
Type III	66.7^a^	40.0	–
Type IV	0.0	2.9	–
Fracture displacement (moderate/severe) (%)	30.6/69.4	34.3/65.7	0.8
Intra-articular extension (%)	66.7/33.3	40.0	**0.03**
Comminution (%)	58.3	40.0	0.16
Time from injury to presentation (days)^b^	3.0 (1.4–4.6)	2.0 (1.1–2.9)	0.83
Time from injury to surgery (days)^b^	4.6 (3.0–6.3)	6.1 (4.3–7.8)	**0.02**
Operative time (min)^b^	97.8 (86.8–108.8)	62.3 (52.3–72.2)	**<0.0001**
Length of follow-up (weeks)^b^	36.6 (22.0–51.2)	19.7 (11.6–27.8)	**0.001**

BMI: body mass index; L: left; R: right.

a One patient with bilateral type III fractures.

b Mean (95% confidence interval).

c N = 25 secondary to missing BMI data for 10 patients.

Bolded p-values are those deemed significant with *p* < 0.05 defined as statistically significant.

The average patient age was 14.6 ± 1.2 years (range, 11.2–17.3 years) (Table 1). Of the 70 patients, 65 (92.9%) were male and 5 (7.1%) were female. The average BMI was 27.8 kg/m^2^ (range, 17.0–39.0 kg/m^2^), while the average age-sex matched percentile BMI was 85 (range, 2–100). Eleven patients (18.3%) were classified as overweight (>85th percentile BMI for age and sex) and 34 (56.7%) were classified as obese (>95th percentile BMI for age and sex). Medical history was significant for three patients with asthma, two with sickle cell trait, and one with sickle cell disease. There was one patient with a recorded history of Osgood-Schlatter. This was the same single patient in our series with bilateral tibial tubercle fractures.

The average time from injury to presentation to our institution was 2.5 days (range, 0–24 days). None of the patients had signs of impending compartment syndrome at presentation. The most common mechanism of injury was a fall (43/70, 61.4%). The most common activity was basketball (18/70, 25.7%), followed by soccer (15/70, 21.4%), general playing (9/70, 12.9%), football (7/70, 10.0%), skateboarding (7/70, 10.0%), non-electric scootering (3/70, 4.3%), physical education class (2/70, 2.9%), hurdles (1/70, 1.4%), running on stairs (1/70, 1.4%), softball (1/70, 1.4%), and baseball (1/70, 1.4%). Five (5/70, 7.1%) did not have a known activity during injury.

Five fractures were classified as type I (5/71, 7.0%), 27 as type II (27/71, 38.0%), and 38 as type III (38/71, 53.5%). One fracture was initially classified type II, but intraoperatively the fracture was found to extend to the posterior tibia and was therefore reclassified as a type IV (1/71, 1.4%). All fractures were displaced, with 23 classified as moderate (23/71, 32.4%) and 48 classified as severe (48/71, 67.6%). Thirty-eight fractures had intra-articular involvement (38/71, 53.5%), while 35 were described as comminuted (35/71, 49.3%).

The average time from injury to surgery was 5.3 days (range, 0–31 days). Overall, the groups were not significantly different with regard to demographic variables (Table 1). Fractures in Group 1 were more likely to be intra-articular (66.7% versus 40.0%, p = 0.03). In addition, there was a greater proportion of type III fractures in Group 1, though this was not statistically significant (66.7% versus 40.0%, p = 0.08). Despite there being no significant difference between the groups in time from injury to presentation (3.0 days versus 2.0 days in Groups 1 and 2, respectively, p = 0.83), fractures in Group 1 underwent surgery sooner after injury than those in Group 2 (4.6 versus 6.1 days, p = 0.02).

The overall average operative time was 77.2 min (range, 25–181 min). The average operative time for fractures in Group 1 (97.8 min) was significantly longer than that for fractures in Group 2 (62.3 min) (p < 0.001). This was true for all fracture types for which groups were large enough to perform statistical analysis (Table 2).

**Table 2.** Operative time by fracture type: inpatient versus outpatient surgery.

	Group 1 (n = 35 patients, 36 fractures)^a^	Group 2 (n = 35 patients, 35 fractures)^b^	p-value
All fractures (min)^c^	97.8 (86.8–108.8)	62.3 (52.3–72.2)	**<0.0001**
Type I (min)^c^	58 (n = 2)^b^	54 (n = 2)	–
Type II (min)^c^	94.9 (65.8–124.0) (n = 9)	59.3 (40.6–78.1) (n = 18)	**0.011**
Type III (min)^c^	99.8 (87.1–112.5) (n = 22)^a^	67.4 (57.4–77.3) (n = 14)	**0.0002**
Type IV (min)^c^	n/a (n = 0)	60 (n = 1)	–

a Excludes operative time for repair of bilateral type III fractures.

b Operative time unavailable for one patient with a type I fracture.

c Mean (95% confidence interval).

Bolded p-values are those deemed significant with *p* < 0.05 defined as statistically significant.

All patients underwent open reduction and internal fixation under fluoroscopic guidance using unicortical cannulated screws. For type III fractures, indirect visualization of the joint surface was gained through the fracture site. No additional arthrotomy was performed. The average number of screws per fracture was 2.2 (range, 1–4 screws), with the most common number of screws placed being 2 (33/71, 46.5%) followed by 3 screws (23/71, 32.4%) and 1 screw (14/71, 19.7%). One patient was treated with four screws (1/71, 1.4%).

Screw configuration by fracture type is outlined in Table 3. Prophylactic fasciotomy was performed for two fractures (2/71, 2.8%), with an average operative time of 102 min. Both were performed early in the study period (Group 1). Both patients were males with type III, severely displaced fractures. There were no confirmed cases of compartment syndrome in either group. Five fractures (5/71, 7.0%) were found intraoperatively to have a concomitant patellar sleeve avulsion requiring repair. Of these five, three were type II fractures (60.0%) and one each were type I (20.0%) and type III (20.0%).

**Table 3.** Screw configuration by fracture type.

	Type I (n = 5)	Type II (n = 27)	Type III (n = 38)	Type IV (n = 1)	Overall (n = 71)	p-value^a^
Number of screws/fracture^b^	1.4 (0.9–1.9)	1.7 (1.5–2.0)	2.6 (2.4–2.8)	1.0	2.2 (2.0–2.4)	**<0.0001**
Screw length^b^	41.4 (37.3–45.5)	37.8 (34.3–41.3)	45.0 (42.7–47.3)	44.0	41.9 (39.9–43.9)	**0.004**
Screw thread (full/partial) (%)	57.1/42.9	63.8/36.2	41.8/58.2	100.0/0.0	49.7/50.3	**0.042**
Screws with washers (%)	85.7	72.3	74.5	100.0	74.5	0.75
Screw location (metaphyseal/epiphyseal) (%)	100.0/0.0	100.0/0.0	42.9/57.1	100.0/0.0	63.4/36.6	–
Number of metaphyseal screws/fracture^b^	1.4 (0.9–1.9)	1.7 (1.5–2.0)	1.1 (1.0–1.2)	1.0	1.4 (1.3–1.5)	**<0.0001**
Number of epiphyseal screws/fracture^b^	0.0 (0–0)	0.0 (0–0)	1.5 (1.3–1.7)	0.0	0.8 (0.6–1.0)	**<0.0001**

a p-value comparing type I, type II, and type III fractures.

b Mean (95% confidence interval).

Bolded p-values are those deemed significant with *p* < 0.05 defined as statistically significant.

Immediately after surgery, 62 (87.3%) patients were placed in a knee immobilizer, 7 (9.9%) were placed straight into a long-leg cast (LLC), and 2 (2.8%) into a long-leg posterior splint. At their first postoperative visit, all patients were placed in an LLC with the exception of the patient who suffered bilateral fractures, for whom an LLC was placed on one side and a knee immobilizer on the other side. The average duration of immobilization was 34 days (range, 15–64 days).

For patients in Group 1, the mean length of stay was 1.4 days (range, 1–6 days (patient with bilateral fractures)). Of patients in Group 1, 28 (78%) spent only one night in the hospital. There was no significant association between fracture type and length of stay (p = 0.85).

The average follow-up time was 27.2 weeks (range, 5.6–191.3 weeks). Patients in Group 1 had significantly longer follow-up time than those in Group 2 (36.6 versus 19.7 weeks, p = 0.001). At final follow-up, all patients had achieved radiographic union and were cleared for full activities, with the exception of three patients who were lost to follow-up (3/71, 4.2%). There were 12 complications totally for an overall complication rate of 16.9%. There was no statistically significant difference in complication rates between the groups (25.0% versus 11.4%, for fractures in Groups 1 and 2, respectively; p = 0.22) (Table 4).

**Table 4.** Complications of tibial tubercle fractures in our series: inpatient versus outpatient surgery.

	Group 1 (n = 36)	Group 2 (n = 35)	p-value
Hardware irritation	1	1	–
Hardware removal	1	1	–
Stiffness	6	1	–
Patellar tendon avulsion	0	1	–
Total complications (% incidence)	8 (25.0)	4 (11.4)	0.22

There were no incidences of compartment syndrome, infection, hardware failure, nonunion, neurovascular injury, or development of growth arrest. One patient with a type II fracture was found at his first postoperative visit to have a patellar tendon avulsion without fracture re-displacement. He was treated with patellar tendon repair using suture anchors and went on to have complete healing. Four patients (4/71, 5.6%) experienced hardware irritation, though only two required hardware removal (2/71, 2.8%). Of fractures with hardware irritation or removal, three were type III and one was type II. There was no significant association between hardware irritation or removal and number of screws (p = 0.32) or number of washers (p = 0.61).

At final follow-up, the mean range of motion was 128° (range, 80°–145°; mean extension 0.3°, mean flexion 128.3°). Patients lacked either full flexion or full extension, but not both. Three patients lacked full extension (extension lag of ≥5°; 3/71, 4.2%) while four lacked flexion (flexion ≤110°; 4/71, 5.6%), for a total of seven fractures classified as having stiffness (7/71, 9.9%). The average duration of immobilization in this group was 31.4 days (range, 15–64 days). There was no association between duration of immobilization and the development of stiffness (p = 0.96). The development of stiffness was not associated with fracture type (p = 0.74), degree of displacement (p = 0.67), intra-articular involvement (p = 1.0), or the presence of comminution (p = 0.71).

## Discussion

Fractures of the tibial tubercle are a relatively uncommon injury for which data on appropriate treatment, complications, and outcomes are limited and heterogeneous. Compartment syndrome is a devastating complication of tibial tubercle fractures, and concern for its development often encourages surgeons to treat these injuries on an emergent, inpatient basis. The purpose of this study was to retrospectively review a large single-surgeon series of operatively treated tibial tubercle fractures in order to better characterize complication rates and to determine the outcome of managing some of these fractures in an ambulatory setting. We hypothesized that, for a selected group of these fractures without preoperative signs of compartment syndrome, there would be no difference in outcomes and complications, specifically compartment syndrome, between fractures treated in an inpatient setting and those treated in an ambulatory setting.

Reported rates of suspected compartment syndrome secondary to tibial tubercle fractures are as high as 10%–20%.^8,23^ As a result, many surgeons elect to perform prophylactic anterior compartment fasciotomy at the time of surgery and admit patients perioperatively for close observation. This study cohort of 71 fractures revealed no cases of compartment syndrome. While prophylactic fasciotomy was performed on two patients, both were early in the treating surgeon’s career and were performed because of high reported rates of compartment syndrome in the literature.

Our institution is unique in that it has its own dedicated pediatric orthopedic urgent care. Many patients are referred to us from outside hospital emergency rooms, urgent cares, or primary care physicians and, consequently, do not present to our institution until days or even weeks after injury; this is reflected on this study’s average time from injury to presentation of 2.5 days (range of 0–24 days). It is very likely that, as a result of this referral pattern, patients who had a massively swollen extremity trending toward a compartment syndrome, or an already established one, were treated elsewhere, skewing our results regarding the possibility of compartment syndrome associated with this injury. This is a very critical point, and one that requires critical judgment by the reader. The ambulatory management of tibia tubercle fracture should be reserved only for patients in whom there are no clinical signs of symptoms of impending compartment syndrome. Any patient with a displaced tibia tubercle fracture associated with a massively swollen extremity or with any sign or symptom of impending compartment syndrome should receive immediate medical attention, including hospitalization, compartment pressure assessment, and surgical decompression when indicated.

By choosing not to this select group of fractures acutely and emergently to the operating room, we were able to confirm that judicious ambulatory management of these injuries is not associated with an increased rate of complications. In addition, this study reveals that the incidence of compartment syndrome may be lower than previously thought, bringing into question the need for prophylactic fasciotomy. It is important to emphasize that the majority of cases of compartment syndrome associated with tibial tubercle fractures occur preoperatively. Indeed, Pretell-Mazzini et al.^2^ revealed a 0% incidence of postoperative compartment syndrome in their systematic review of 336 fractures. Therefore, in the absence of signs of preoperative compartment syndrome, we believe these fractures can be safely treated in a delayed, ambulatory manner. Ideally, these fractures should be treated within 14 days to optimize ease of surgery as significant macroscopic bony callus has been shown to appear as soon as 4 weeks after injury in animal models.^29^

For those procedures in which it is safe to do so, ambulatory orthopedic procedures provide a benefit in the form of increased efficiency, fewer complications, and lower costs.^30–32^ Recent studies have confirmed that these benefits of ambulatory procedures also apply to pediatric orthopedic surgery.^12–14^ In our study, average operative times for surgeries performed on an ambulatory basis were significantly shorter overall than those performed on an inpatient basis, despite the same surgeon performing the same procedure in both settings without significantly different fracture characteristics between the two groups (Table 2). Given the importance of limiting anesthesia time and hospital exposure in pediatric patients, this finding is of particular significance in this population.

This study echoes prior literature that demonstrates unicortical fixation is sufficient for treating tibial tubercle fractures.^10^ All patients in our cohort were treated with unicortical, cannulated screws with adequate fixation and healing. There was no need to maximize fixation with bicortical screws and risk major vessel injury about the knee.^10^ The number and configuration of screws should be determined by fracture type. Type I and II fractures can be successfully treated with 1–2 metaphyseal screws, while 1–2 epiphyseal screws should be added when managing type III fractures. Reported rates of symptomatic hardware removal are varied, ranging from 4.4%^10^ to as high as 74%.^5,27^ While our study revealed a 5.6% incidence of hardware irritation and a 2.8% incidence of hardware removal, this complication was not associated with any specific hardware-related factors such as number of screws or washers used. Therefore, these surgical considerations should be made on an individual case-by-case basis centered on patient-specific factors such as fracture morphology, degree of comminution, and bone quality.

This study demonstrated a 5.6% incidence of stiffness, which is higher than rates reported in the literature.^2,10^ However, in their systematic review, Pretell-Mazzini et al.^2^ demonstrated a mean return to full range of motion of 22.3 weeks. Of the seven patients in our study classified as having stiffness, only one was followed for more than this length of time, while the remainder were lost to follow-up or discharged from care after a range of 6–17 weeks. This discrepancy in follow-up time likely explains the higher incidence of stiffness seen in our study cohort. Overall, duration of immobilization was not associated with the development of stiffness, nor where there any fracture characteristics found to be predictive. Given these findings, type and duration of postoperative immobilization should prioritize fracture healing over range of motion, especially given long-term studies have demonstrated excellent functional outcomes in the overwhelming majority of fractures.^16^

There are several limitations of this study, including the retrospective nature of the review. Average follow-up time was relatively short at only 7 months. However, all but three patients who were lost to follow-up had achieved radiographic healing and had been cleared for full activity at their final visits. Follow-up time was significantly longer in the inpatient group compared with the ambulatory group. This can be explained by the opening of a new freestanding Ambulatory Surgery Center (ASC) at our institution in 2016. Prior to this time, more fractures were treated at our inpatient hospital secondary to operating room availability. Therefore, most inpatient fractures were treated early in the study cohort and as a result have longer follow-up times. While this could theoretically result in missed long-term sequelae of tibial tubercle fractures in the ambulatory group, the main focus of this study was the immediate safety of ambulatory management and it was therefore outside the scope of our analysis to comment on long-term outcomes.

The decision of inpatient versus ambulatory management was at the discretion of the treating surgeon, introducing a possibility of selection bias and confounding, with more severe injuries being managed in the inpatient setting. This may explain the higher proportion of type III fractures seen in the inpatient group, though this was not statistically significant. We believe this is more likely explained by a skew toward more type III fractures being treated early in the study period, prior to the opening of a new ASC in 2016. There is also a notable transition period in 2014–2016 that coincided with increased experience and comfort of the treating surgeon with ambulatory management while still limited by lack of ready availability of an ASC. Four of the 20 fractures treated during this time period stayed at least one night in the hospital. All were type III fractures with moderate to severe displacement, and two of them were comminuted, which may have contributed to the decision for an inpatient stay. All but one patient presented 2–7 days after injury, so there was less concern about significant postoperative compartment syndrome. Average surgical time did not differ compared to the rest of the series (79 min). However, three of the four surgeries ended after 5 pm, and one surgery ended after 8 pm due to operating room availability constraints versus time of presentation at our institution. Later surgery end times likely contributed to overnight stays due to concerns of availability of adequate pain control and late shift hospital support staff to manage pediatric patient discharges at our institution. This intermittent lack of dedicated support staff contributed to our institutions’ desire to open up a dedicated pediatric orthopedic ASC. Although our study cohort included 14 type III fractures treated in the ambulatory setting with only 2 minor complications—hardware irritation and stiffness—we do not advocate that all tibial tubercle fractures should be managed in the ambulatory setting, and there are certainly indications to admit patients for perioperative observation. Therefore, it is important that the operating surgeon maintain privileges at a nearby hospital should issues arise intraoperatively or postoperatively that warrant inpatient admission for observation.

Despite these shortcomings, this is the largest single-surgeon study to our knowledge to examine the safety of ambulatory surgical management of tibial tubercle fractures. We demonstrated that ambulatory management had no difference in complication rates and significantly faster surgical time when compared to inpatient management. By limiting patients to those treated by a single surgeon, treatment method, surgical technique, and postoperative protocol were standardized across both the inpatient and ambulatory setting. Despite the unique nature of our institutions’ dedicated pediatric orthopedic urgent care and ambulatory surgery center, our study population echoed the typical demographic described in the literature, with an average age of 14.6 years and a 93% male predominance.^2,10,15,16,33^ Given these similarities, we feel our study cohort is an accurate reflection of the general population.

In conclusion, patients with operatively treated tibial tubercle fractures have excellent radiographic and functional outcomes and low complication rates regardless of fracture type or surgical setting. In addition, the risk of compartment syndrome may be lower than previously described, warranting consideration for ambulatory management of appropriately indicated patients. However, the ambulatory management of tibia tubercle fracture should be reserved only for patients in whom there are no clinical signs of symptoms of impending compartment syndrome at the time of presentation. Any patient with a displaced tibia tubercle fracture associated with a massively swollen extremity or with any sign or symptom of impending compartment syndrome should receive immediate medical attention, including hospitalization, compartment pressure assessment, and surgical decompression when indicated.

## Supplemental Material

sj-pdf-1-cho-10.1177_18632521231214317 – Supplemental material for Ambulatory surgical management of most displaced tibial tubercle fractures in children is safe and efficient Click here for additional data file. Supplemental material, sj-pdf-1-cho-10.1177_18632521231214317 for Ambulatory surgical management of most displaced tibial tubercle fractures in children is safe and efficient by Brian Kendrick Zukotynski, Danielle Brown, Kellyn Hori and Mauricio Silva in Journal of Children’s Orthopaedics
